# Prevalence of human papillomavirus infection among women from quilombo communities in northeastern Brazil

**DOI:** 10.1186/s12905-017-0499-3

**Published:** 2018-01-02

**Authors:** Maria do Desterro Soares Brandão Nascimento, Flávia Castello Branco Vidal, Marcos Antonio Custódio Neto da Silva, José Eduardo Batista, Maria do Carmo Lacerda Barbosa, Walbert Edson Muniz Filho, Geusa Felipa de Barros Bezerra, Graça Maria de Castro Viana, Rebeca Costa Castelo Branco, Luciane Maria Oliveira Brito

**Affiliations:** 1 Department of Pathology, Center of Biological and Health Sciences, Federal University of Maranhão, São Luís, Brazil; 2 Department of Morphology, Center of Biological and Health Sciences, Federal University of Maranhão, São Luís, Brazil; 3 Federal University of Maranhão, São Luís, Brazil; 4 Post-graduate Program in Adult and Child Health, Federal University of Maranhão, São Luís, Brazil; 5 Department of Medicine I, Center of Biological and Health Sciences, Federal University of Maranhão, São Luís, Brazil; 6 Department of Medicine III, Center of Biological and Health Sciences, Federal University of Maranhão, São Luís, Brazil; 7 Núcleo de Imunologia Básica e Aplicada, Avenida dos Portugueses, 1966, Bacanga. Prédio do CCBS, Bloco 3, Sala 3A, São Luís – MA, CEP 65080-805 Brazil

**Keywords:** Human papillomavirus (HPV), Prevalence, Risk factors, Genotypes, Cervical cancer, Pap smear, Quilombola women

## Abstract

**Background:**

Human papillomavirus (HPV) is a member of the *Papillomaviridae* family. The prevalence of HPV genotypes may vary according to the region and the population studied. Quilombo communities are ethnic and racial groups with difficult access to health services compared to the general population in Brazil. The aim of this study was to identify specific HPV types correlating with sociodemographic/behavioral characteristics and cervical smear cytological abnormalities in Quilombola women.

**Methods:**

This cross-sectional study included 395 Quilombola women users of the Unified Health System of the Municipalities of Maranhão for the screening of cervical cancer. The samples were analyzed for the presence of cytological abnormalities by conventional methods and tested for 37 HPV genotypes using polymerase chain reaction with primers PGMY09/11 followed by reverse line blot hybridization performed with the *Linear Array HPV Genotyping Test* kit by Roche Molecular System®. The association between HPV types and cytological diagnosis was investigated according to the different age groups.

**Results:**

HPV infection was detected in 12.6% (50/395) of the women. Infections by high-risk HPV types were more frequent. Genotypes 68 (26.0%); 58 and 52 (20.0%); 31 (10.0%) and 62 (8.0%) were the most prevalent. The highest prevalence (42.0%) of HPV infection occurred in women diagnosed with high-grade squamous intraepithelial lesion. There was a statistically significant association between HPV infection and the detection of cytological abnormalities in all age groups except in women over 60 years. There was a statistically significant association between the municipality of origin and the number of partners with HPV infection.

**Conclusions:**

It is important to incorporate new cervical cancer screening techniques incorporating the cervical-vaginal cytology. For further studies, it is necessary to determine the level of knowledge of Quilombola population on health-related issues including HPV infection and cervical cancer. This will contribute to the continuous improvement of healthcare coverage among the population and enhance the implementation of cancer care in the state of Maranhão.

## Background

Cervical cancer (CC) is the third most common cancer among women worldwide, with approximately 528,000 new cases per year, being responsible for the death of 266,000 women each year [1]. According to the Brazilian National Cancer Institute, the estimated number of cases of CC for the year 2014 (and 2015), were approximately 15,590 in Brazil. CC is the second most common neoplasm in the northeast Brazil (18.79 cases/100,000 inhabitants). In Maranhão, annually, 880 new cases are estimated and 200 new cases in São Luís, thus surpassing breast cancer [2]. The prevalence of HPV in Brazil ranges from 16.8% to 26.8% in women not stratified by cytology [3, 4]. In a study on indigenous populations, the prevalence of HPV by polymerase chain reaction (PCR) and hybrid capture was 29.0%. [5].

Human papillomavirus (HPV) belongs to the *Papillomaviridae* family, genus *Papillomavirus*. It is an icosahedral virus, non-enveloped, and about 55 nm in diameter. The genome consists of a double-stranded circular DNA, containing approximately 7900 base pairs [6]. Thus far, about 200 HPV types (designated with the HPV acronym and an assigned number, as characterized) have been identified [7].

HPVs are classified into low or high oncogenic risk groups, according to their ability to cause malignant lesions including cervical carcinomas. The low-risk types (HPV types: 6, 11, 40, 42, 43 and 44) are associated with the development of benign lesions such as warts, while the high risk types (HPV types: 16, 18, 31, 33, 35, 39, 45, 51, 52, 56, 58, 59 and 66) are associated with the process of carcinogenesis. HPV types 16 and 18 are related to more than 70% of CC cases [8-10].

HPV types 31, 33, 35, 45, 52 and 58 are the most common, but there are large variations in the frequencies [11]. For example, in developed countries, HPV 58, HPV 33 and HPV 45 are listed as the third, fourth, and fifth most prevalent HPV types respectively, when associated with cancer. Among the developing countries, HPV 33, HPV 31, and HPV 45 hold these respective positions [12]. It is estimated that most men and women are infected with at least one subtype of HPV during their sexual life, although with most of the people, the infection neither persists nor progresses to cancer [13].

Persistent HPV infection is a necessary condition for the development of intraepithelial preneoplastic lesions and invasive CC. However, it was demonstrated that, by itself, HPV is not a sufficient cause. HPV requires the association of other co-factors for the development, maintenance, and progression of intraepithelial lesions [14–16]. Several studies have evaluated the risk factors for genital HPV infection. Such risk factors include, the number of sexual partners, early sexual activity, new sexual partner, smoking, prolonged use of oral contraceptives, history of sexually transmitted diseases (STDs) including infection by *Chlamydia trachomatis*, herpes simplex type 2 and HIV, as well as factors such as immunosuppression and genetic predisposition [17].

The earliest changes of cervical lesions are low-grade cytological abnormalities such as atypical squamous cells of undetermined significance (ASC-US) and low-grade squamous intraepithelial lesion (LSIL). A systematic review of 423 studies showed that HPV-DNA from 48 different types of alpha-papillomavirus HPV was present in 52.1% of ASC-US and 74.2% of LSIL lesions [18]. A meta-analysis of 32 studies reported that high-risk HPV were detectable in 43% of ASC-US and in 76% of cervical intraepithelial neoplasm grade 1 [19].

Currently, two virus-like particles-based vaccines formed by L1 proteins of HPV 6, −11, −16, and 18 (Gardasil) or HPV 16 and −18 (Cervarix) are available on the market. Both proved to be highly immunogenic vaccines in clinical trials, resulting in 100% seroconversion in the different studied populations [20, 21].

According to information from the Federation of Associations of the State of Maranhão Municipalities (FAMEM) [22], Maranhão is the Brazilian state with the largest number of Quilombo remnants. The are 642 Quilombo communities, with only 369 recognized by the Palmares Cultural Foundation and the Federal Government with publications in the Official Gazette [23]. Quilombola communities are ethnic and racial groups according to auto-allocation criteria, with its own historical background [24]. This history entailed geographical isolation with specific territorial relations, the presumption of black ancestry, monogamous relationships (only between members of Quilombos), and with resistance to historical oppression [24]. Ethnic and racial disparities are seen in CC. Non-Hispanic black women and Hispanic women have higher incidence and mortality rates than other racial groups [25].

The data about the health of the Quilombola population are scarce, especially in relation to prevalence, genotypes, and risk factors for HPV infection. Therefore, data about the distribution of specific types of HPV in the region and their relationship to the carcinogenic potential of HPV in the diagnosis of associated cytologic abnormalities are important tools to evaluate the behavior of these infections and associated lesions in the post-vaccination period.

The aim of this study was to determine the prevalence and the distribution of HPV genotypes in Quilombola women and to evaluate the risk factors associated with HPV infection.

## Materials and methods

### Type and area of study

This was a cross-sectional study carried out to determine the prevalence of CC and HPV infection in Quilombola women from the State of Maranhão, in the Municipalities of São José de Ribamar, Presidente Vargas, Viana, São Luís Gonzaga, Central do Maranhão and Alcântara. The studied Quilombola communities were Juçatuba (São José de Ribamar); Sapucaia, Sororoca, Boa Hora I, Boa Hora II, Cavianã, Cigana, Estiva dos Cotos, Irmã Dorate, Lagoa Grande, Marajá, Pau D’arco and Fincapé (Presidente Vargas); São Cristóvão, Rua Grande, Rua Linares Pinheiros, Rua Santa Luzia and Rua Principal (Viana); Café Pipira, Monte Cristo, Fazenda Velha, Natal, Vale Verde, Santo Antônio da Costa and Antonio Matos (São Luís Gonzaga); São Sebastião (Central do Maranhão) and Mocagituba I, Mocagituba II, Cajueiro, Santo Inácio, Povoado Oitiva, Povoado Castelo, Povoado Raimundo Sú, Novo Belém, Povoado Lisboa, Tubarão and Goiabal (Alcântara).

### Study population

The population selected for this study consisted of 161 Quilombola families (being the sample size estimated for 395 people), aged between 12 and 84 years, recruited from March 2012 to February 2013.

The inclusion criteria were: Quilombola women who have had or still have sexual activities and who spontaneously sought the Cervical Cancer Control Program examination as users of the Unified Health System of the Municipalities of Maranhão, and who signed the free and informed consent term.

Exclusion criteria: hysterectomized Quilombola women, or those subjected to cervical surgery, those with mental deficit (this might undermine their understanding of the questions as well as the answers to complete the specific forms), and women whose samples were with DNA degradation or with unsatisfactory vaginal cervical smear.

### Study sample

The study sample consisted of 395 Quilombola women who underwent cytological examination, PCR and genotyping for HPV detection.

### Data collection

The sample collection was carried out from March 2012 to February 2013. It was preceded at the time of sample collection by an interview using a structured epidemiological questionnaire that included information on sociodemographic characteristics, habits, as well as sexual and reproductive history.

The women underwent careful clinical examination with an inspection of the external genital and perianal region. Secretions of ectocervix and endocervix, taken for cytological examination, were conducted by conventional method. The biologic samples were also immersed in a preserving buffer media and used for HPV-DNA detection by PCR [26] and genotyping by reverse hybridization the Linear Array HPV Genotyping Test kit (Roche Molecular Systems). The samples were stored in a freezer at −20 °C until DNA extraction (in the Biobank of tumors and DNA of the Federal University of Maranhão (UFMA).

### Cytological examination

Cytological smears consisted of ectocervical and endocervical mucous samples (squamo-columnar junction), collected with Ayre’s spatula and endocervical brush. The cell smear was held in a glass slide, which was fixed with alcohol and forwarded for staining using the Papanicolau technique at the Nucleus of Basic and Applied Immunology, Department of Pathology of UFMA. All smears were subjected to a strict quality control adopted by the laboratory. This was done by reviewing all negative smears for cervical cytological abnormalities by three cytopathologists. The results of suitability of the samples and the degree of cervical abnormalities were interpreted according to the Bethesda System revised in 2001 [27].

### HPV analysis

The extraction of the genomic DNA from the samples was performed using the QIAamp DNA FFPE Tissue Purification Kit (QIAGEN®) according to the extraction protocol suggested by the manufacturer.

The Nested PCR reactions were performed by using primers PGMY09 and PGMY11 for the first round, and primers GP + 5 and GP + 6 for the second round [26, 28].

After denaturation, the material was applied to a nylon strip containing immobilized probes for two different concentrations of β-globin, 19 HPV genotypes with high oncogenic risk (16, 18, 26, 31, 33, 35, 39, 45, 51, 52, 53, 56, 58, 59, 66, 68, 69, 73 and 82) and 18 types of HPV with low oncogenic risk (6, 11, 40, 42, 54, 55, 61, 62, 64, 67, 70, 71, 72, 81, 83, 84, IS39 and CP6108).

### Statistical analysis

The data were analyzed using the statistical program SPSS for Windows 20.0 (SPSS Inc., Chicago, USA). Initially, exploratory (descriptive) analyzes of the numerical variables (age, age at menarche and the onset of sexual activity), calculating the maximum, minimum, medians and standard deviation were done. Association between HPV types and cytology results for women with single infection was performed with the chi-square test. To evaluate the association between HPV types and sociodemographic and clinical variables, a nonparametric ANOVA was performed using the Kruskal Wallis test. In all tests, the significance cut off level (α) was 5% and the results were considered significant when *p* < 0.05.

### Ethical aspects

This project was submitted and approved by the institutional review board (IRB) of the University Hospital of Federal University of Maranhão and the IRB of the Federal University of Maranhão. To participate, the women signed the free and informed consent term. For the minors/children enrolled in this study, the objectives were explained to them and they signed the agreement consent (Resolution 466/12 of the National Health Council and its complementary).

## Results

A total of 395 women were included in the study, with 27.8% (110) being less than 30 years of age. The age of patients ranged from 12 to 84 years, with a mean age of 41.3 ± 14.4 years. Most women, 261 (66.0%) had finished elementary school, were married, 241 (61.0%) and by race, most, 180 (45.5%) were brown (Table 1).

**Table 1:** Sociodemographic and behavioral characteristics of Quilombola women

Variables	N	%
Age group		
≤ 30	110	27.8
31–40	97	24.6
41–50	80	20.3
51–60	69	17.5
>60	39	9.9
Education		
Illiterate	60	15.2
Elementary School	261	66.0
High School	65	16.5
Higher Education	9	2.3
Marital Status		
Married	241	61.0
Other	154	39.0
Race		
White	26	6.6
Black	128	32.4
Brown	180	45.5
Not informed	61	15.5
Beginning of sexual activity		
≤ 15 years	152	38.4
> 15 years	243	61.6
Number of partners		
One	179	45.3
More than one	216	54.7
Age of Menarche		
≤12 years	162	41.0
>12 years	233	59.0
N° pregnancies		
Up to one	69	17.5
More than one	326	82.5
STD Signs		
No	278	70.4
Yes	117	29.6
Use of contraceptive		
Yes	70	17.7
No	325	82.3
Genital hygiene frequency		
Once a day	70	17.7
More than once a day	325	82.3
Performing of Papanicolau examination		
No	58	14.7
Yes	337	85.3
Inspection of the Cervix		
Altered	192	48.6
Normal	203	51.4
Alcoholic		
Yes	145	36.7
No	250	63.3
Smoker		
Yes	32	8.1
No	363	91.9

Table 1 shows the behavioral characteristics of the participants. The minimum and maximum age at menarche was 9 years and 17 years respectively, with a median age of 13 years ±1.5 years. The earliest sexual initiation was by 10 years, with only case of sexual initiation by 43 years of age, with a median of 16.6 years ±3.1.

Among the 395 women, 33 (8.4%) had cytological abnormalities, which included 5 (15.2%) women with ‘atypical squamous cells, cannot exclude high-grade squamous intraepithelial lesion [HSIL]’ (ASC-H), 11 (33.3%) women with ‘ASC-US, 10 (30.3%) women with HSIL, and 7 (21.2%) women with LSIL.

The overall prevalence of HPV was 12.6% (50/395). In Table 2, among the 33 women who had cytological abnormalities, 12 (36.4%) were positive for HPV. From the 395 women, studied, 336 had diagnoses of inflammation, out of which 35 (10.4%) were positive for HPV. For ASC-H, ASC-US, HSIL and LSIL, HPV infection rate were 40.0% (2/5), 18.2% (2/11), 60.0% (6/10) and 28.2% (2/7) respectively (Table 2). There was a statistically significant association between the presence of cytological abnormalities in cervical smears and the presence of HPV (*p* < 0.001).

**Table 2:** HPV prevalence by cytologic abnormalities in Quilombola women

	HPV			
Cytological Abnormalities	Negative n (%)	Positive n (%)	Total n (%)	p
ASC-H	3 (60.0)	2 (40.0)	5 (100.0)	<0.0001
ASC-US	9 (81.8)	2 (18.2)	11 (100.0)	
HSIL	4 (40.0)	6 (60.0)	10 (100.0)	
LSIL	5 (71.4)	2 (28.6)	7 (100.0)	
Inflammation	301 (89.6)	35 (10.4)	336 (100.0)	
Atrophy	23 (88.4)	3 (11.6)	26 (100.0)	
Total	345 (87.4)	50 (12.6)	395 (100.0)	

ASC-H - (atypical squamous cells cannot exclude high-grade squamous intraepithelial lesions)

ASC-US - (atypical squamous cells of undetermined significance)

HSIL - (high-grade intraepithelial lesion)

LSIL - (low-grade squamous intraepithelial lesion)

Among the HPV types, the most prevalent genotypes were 68 (26.0% - 13/50), 58 and 52 with 20.0% (10/50) each, 31 (10.0% - 5/50) and 62 (8.0% - 4/50). Only 6.0% (3/50) of the cases were positive for HPV-61 (Table 3).

**Table 3:** Distribution of HPV genotypes in the cytologic findings of Quilombola women

HPV Types	Cytological Abnormalities						
	ASC-H	ASC-US	HSIL	Inflammation	LSIL	Atrophy	Total
HPV High risk	n (%)	n (%)	n (%)	n (%)	n (%)	n (%)	n (%)
16	–	–	1	–	–	–	1
18	–	–	1	1	–	–	2
31	–	–	–	4	1	–	5
33	–	–	–	1	–	–	1
39	–	–	–	1	–	–	1
45	–	–	–	2	–	–	2
51	–	–	–	1	–	–	1
52	–	1	2	6	–	1	10
56	–	–	–	1	–	–	1
58	–	1	2	6	–	1	10
59	–	–	–	–	–	1	1
66	–	–	–	1	–	–	1
68	–	1	–	11	–	1	13
70	–	–	–	2	–	–	2
73	1	–	–	–	–	1	2
HPV Low risk							
53	–	–	–	2	–	–	2
54	–	–	–	2	–	–	2
55	–	–	–	1	–	–	1
61	–	–	–	2	1	–	3
62	–	–	–	3	1	–	4
71	–	–	–	–	–	1	1
72	–	–	–	1	–	–	1
84	–	–	1	–	–	–	1
IS39	1	–	1	–	–	–	2
CP6108	–	–	–	–	–	1	1
Total of positives	2	3	8	49	3	7	71*

*This number refers to simple and multiple infections. Among 6 multiple infections of the virus 68, 3 were associated with the virus type 52

ASC-US, atypical squamous cells of undetermined significance; LSIL: low-grade squamous intraepithelial lesion; ASC-H: atypical squamous cells cannot exclude high-grade squamous intraepithelial lesion; HSIL: high-grade squamous intraepithelial lesion

Regarding the presence of HPV in different cytologic findings, the high oncogenic risk type were more frequent (78.0%, 39/50) compared with the low oncogenic risk type (22.0% -11/50). Among those diagnosed with an abnormality, the prevalence of HPV infection was 24.0% (12/50). The highest prevalence was reported with HSIL (42.0%), followed by LSIL (21.0%) and ASC-US (21.0%). In all diagnoses, simple infections were more frequent than multiple (Table 3).

Table 4 shows the prevalence of HPV in women with different cytologic findings, stratified by age group. In women younger than 30 years, positivity for HPV ranged from 23.2% (25/108) in women with inflammatory smears to 100% in women with LSIL and HSIL findings. There was a significant association between HPV positivity and cytologic abnormalities detection in these age groups: younger than 30 years (*p* = 0.02), 31–40 years (*p* = 0.0009), 41 to 50 years (*p* = 0.03) and 51 and 60 years (*p* = 0.0003). Only among HPV-positive women, aged over 60 years, were there no statistically significant association with the detection of cytological abnormalities (*p* = 0.12).

**Table 4:** HPV prevalence by cytologic findings and according to age groups in Quilombola women

	HPV Detection		p
Cytologic Finding	Negative n (%)	Positive n (%)	
Age group (<30 years)			
ASC-US	3 (60.0)	2 (40.0)	0.02
HSIL	–	2 (100.0)	
LSIL	–	2 (100.0)	
Inflammation	83 (76.8)	25 (23.2)	
Atrophy	2 (100.0)	–	
Total	88 (74.0)	31 (26.0)	
Age group (31–40 years)			
ASC-H	–	2 (100.0)	0.0009
ASC-US	2 (100.0)	–	
HSIL	1 (100.0)	–	
LSIL	2 (50.0)	2 (50.0)	
Inflammation	77 (84.6)	14 (15.4)	
Atrophy	–	2 (100.0)	
Total	82 (80.4)	20 (19.6)	
Age group (41–50 years)			
ASC-H	2 (100.0)	–	0.03
ASC-US	2 (66.7)	1 (33.3)	
HSIL	–	2 (100.0)	
LSIL	1 (100.0)	–	
Inflammation	62 (85.0)	11 (15.0)	
Atrophy	1 (50.0)	1 (50.0)	
Total	68 (82.0)	15 (18.0)	
Age group (51–60 years)			
ASC-H	–	1 (100.0)	0.0003
ASC-US	1 (100.0)	–	
HSIL	1 (20.0)	4 (80.0)	
Inflammation	49 (90.7)	5 (9.3)	
Atrophy	11 (73.3)	4 (26.7)	
Total	62 (81.5)	14 (18.5)	
Age group (>60 years)			
ASC-US	–	1 (100.0)	0.12
LSIL	1 (100.0)	–	
Inflammation	19 (82.6)	4 (17.4)	
Atrophy	10 (90.9)	1 (9.1)	
Total	20 (80.0)	5 (20.0)	

ASC-US, atypical squamous cells of undetermined significance; LSIL: low-grade squamous intraepithelial lesion; ASC-H: atypical squamous cells cannot exclude high-grade squamous intraepithelial lesion; HSIL: high-grade squamous intraepithelial lesion;

In Table 5, with sociodemographic variables, only the municipality of origin showed significant association with HPV positivity (*p* < 0.0001). The remaining sociodemographic variables (age, education, marital status, and race) showed no significant association with HPV positivity.

**Table 5:** Univariate analysis of sociodemographic factors associated with HPV infection in Quilombola women

	Detection of HPV		p
Variables	Negative n (%)	Positive n (%)	
Age			
≤30 years	91 (82.7)	19 (17.3)	0.65
31 to 40 years	87 (89.7)	10 (10.3)	
41 to 50 years	72 (90.0)	08 (10.0)	
51 to 60 years	60 (86.7)	09 (13.3)	
>60 years	35 (89.7)	04 (10.3)	
Education			
Illiterate	51 (85.0)	09 (15.0)	0.72
Elementary School	231 (88.5)	30 (11.5)	
High School	56 (86.1)	09 (13.9)	
Higher Education	7 (77.7)	02 (22.3)	
Origin			
Alcântara	96 (95.0)	05 (5.0)	<0.0001
Central do Maranhão	45 (90.0)	05 (10.0)	
Presidente Vargas	46 (73.0)	17 (27.0)	
São José de Ribamar	91 (90.0)	10 (10.0)	
São Luís Gonzaga	40 (74.0)	14 (26.0)	
Viana	25 (96.1)	01 (3.9)	
Marital status			
Single	100 (84.0)	19 (16.0)	0.24
Married	216 (89.6)	25 (10.4)	
Divorced	05 (83.3)	01 (16.7)	
Widow	24 (82.7)	05 (17.3)	
Race			
White	21 (80.7)	05 (19.3)	0.57
Black	109 (85.1)	19 (14.9)	
Brown	159 (88.3)	21 (11.7)	
Not informed	56 (91,.8)	05 (8.2)	

The behavioral predictors of HPV, at the univariate level, are shown in Table 6. None of the variables analyzed (onset of sexual activity, number of partners, age at menarche, number of pregnancies, STD signs, use of contraceptive, hygiene frequency, performing of Pap test, result of the inspection of the cervix, drinking and smoking), showed significant association with HPV positivity except the number of partners (*p* = 0.01).

**Table 6:** Univariate analysis of behavioral factors associated with HPV infection in Quilombola women

Behavioral variables	Detection of HPV		p
	Negative n (%)	Positive n (%)	
Onset of sexual activity			
≤15 years	126 (82.9)	26 (17.1)	0.65
>15 years	219 (90.1)	24 (9.9)	
Number of partners			
One	158 (88.2)	21 (11.8)	0.01
More than one	187 (86.5)	29 (13.5)	
Age at menarche			
≤12 years	141 (87.0)	21 (13.0)	0.22
>12 years	204 (87.5)	29 (12.5)	
Number of pregnancies			
Up to one	63 (91.3)	06 (8.7)	0.32
More than one	282 (86.5)	44 (13.5)	
STD Signs			
No	234 (84.1)	44 (14.9)	0.56
Yes	111 (94.8)	06 (5.2)	
Use of contraceptive			
Yes	53 (75.7)	17 (24.3)	0.47
No	292 (89.8)	33 (10.2)	
Frequency of genital hygiene			
Once	58 (82.8)	12 (17.2)	0.30
More than once	287 (88.3)	38 (11.7)	
Performing of Pap test			
No	49 (84.4)	09 (15.6)	0.17
Yes	296 (87.8)	41 (12.2)	
Inspection of the Cervix			
Altered	169 (88.0)	23 (12.0)	0.59
Normal	176 (86.7)	27 (13.3)	
Alcohol			
Yes	127 (87.6)	18 (12.4)	0.99
No	218 (87.2)	32 (12.8)	
Smoker			
Yes	25 (78.1)	07 (21.9)	0.98
No	320 (88.1)	43 (11.9)	

## Discussion

In Brazil, the Black population experience higher cancer mortality rates compared to the general population. This is probably a consequence of inequities in the social, economic, political, and health factors. This population is faced with unequal experiences at birth, in life, during illness and in death [29]. Quilombo communities fall within this context, as they are presumed to have Black ancestry, and they are also presumed to have important social risk experiences such as the historical process of expropriation of culture and rights. This led to social and health inequality, the impact of which is reflected in poor health indicators among this population [30]. Since the non-realization of the importance of Pap smear examination is associated among other things with the inequalities in the access to and the utilization of health services, it is important to consider the preventive measures for CC as requiring the improvement of the living conditions and the increased supply and access to health services in Quilombo communities [29].

The overall prevalence of HPV can vary depending on the technique used, as well as the population and the region studied. In this study, the prevalence of HPV infection in Quilombola women was 12.6% by hybridization technique. Different results were found in various studies including Akarolo-Anthony et al. (2014), which showed an overall prevalence of 37% in Nigerian women [31], and Watson-Jones et al. (2013), with a prevalence of 74% among women in Tanzania [32]. Cervantes et al. (2003), in a study on indigenous peoples of the Bolivian Amazon, found a prevalence of 5.9% of HPV DNA [33]. Pinto et al. (2011) investigated the prevalence and risk factors for genital infection with HPV in women from rural and urban areas in two different regions of the Eastern Brazilian Amazon and found an overall HPV infection prevalence of 14.6% (15.0% in urban and 14.2% in rural women), similar to our study [34].

Some women had multiple HPV infections in this study, and these occurred more frequently with HPV 68, with a total of 71 HPV types among HPV positive women, and among these, 50 (69.4%) were of high oncogenic risk. Of the 25 HPV types found in this study, HPV 68 was the most prevalent (26.0%), followed by HPV 58 and 52 (20.0%) and HPV 31 (10.0%).

A study conducted in France showed HPV 16 to be most prevalent, followed by HPV 53 and 31 [35]. Study in Vietnam showed the HPV types 16, 18 and 58 as most prevalent [14]. Xue et al. (2015) found that HPV types 16, 52 and 58 were more prevalent, while HPV 68 was only the eighth most prevalent [36].

Goldman et al. (2013) observed that the presence of HPV 68 occurred at a rate four times higher, than expected, in multiple infections. Thus, this indicates the existence of an interaction between HPVs of high oncogenic risk, especially between types 31/68, 51/68 and 33/58. These authors suggested that some virus genotypes act as co-factors in the infection by other types, demonstrating a new possible risk factor [37].

Considering the prevalence of HPV in women with different cytologic findings in this study, age group (particularly less than 30 years) showed important influence on HPV positivity and the detection of cytologic abnormalities. Thus, these results indicate that HPV testing is useful in women over 30 years, who are more likely to be carriers of significant cervical lesions. According to Akarolo-Anthony et al. (2014), the age group most affected by HPV infection were those aged less than 30 years, while a decline were shown in women over 45 years of age, thus corroborating the findings of this study [31]. In Fiji, in the studied population, with age, a decline of HPV infection rates were shown, and these ranged from 35.8% among women aged less than 25 years to 18.6% in women aged 55–64 years [38].

In a study by Foliaki et al. (2014), the positivity for at least one HPV genotype was 24.0% among 1244 women studied, however, HPV 16 was the most prevalent genotype, while HPV 68 was present in only 3 (0.2%) of the infected women. From the HPV positive cases, 70.1% had only one viral genotype, while 29.9% had multiple infections. HPV genotypes most often involved in multiple infections were of the high oncogenic risk [38].

Among the 33 women who had cytological abnormalities, 36.4% were HPV positive. Among those diagnosed with inflammation, 10.4% were HPV positive. For ASC-H, ASC-US, HSIL and LSIL, HPV infection rate were 40.0%, 18.2%, 60.0% and 28.2% respectively. There was a statistically significant association between the presence of cytological abnormalities in cervical smears and the presence of HPV (*p* < 0.001). Marks et al. (2015) found a much higher prevalence (43.8%) of HPV infection among women diagnosed with inflammation, 36.5% diagnosed with ASC-US, 80.9% of women diagnosed with LSIL and 71.5% of women with the diagnosis of HSIL [39]. The HPV type 68, in single or multiple infections, was detected mainly in women whose smears were considered inflammatory. The high-risk types of HPV 58, 52, 18 and 16 were detected in Quilombola women with cytological diagnosis of HSIL, agreeing with other findings in the literature [35, 39, 40].

Based on the presence of HPV in different cytologic findings, the high oncogenic risk types were more frequent (78.0%) than those of low oncogenic risk (22.0%). This has important implications for the clinical care of women with the high oncogenic risk. For those diagnosed with an abnormality, the prevalence of HPV infection was 24.0%, and was highest with HSIL (42.0%).

In relation to sociodemographic factors and HPV infection, only the municipality of origin was significantly associated with positivity for HPV (*p* < 0.0001). Akarolo-Anthony et al. (2014), in studying Nigerian women, also found no association between sociodemographic factors (education, age, marital status) and HPV infection [31]. This may be related to the characteristics of the study population including, geographical isolation, conservative habits as well as non-consumption of alcohol and tobacco.

In this study, there was significant association with HPV positivity only in relation to the number of partners (*p* = 0.01). Work carried out in Guinea by Keita et al. (2009) also demonstrated a significant association between the number of partners and HPV infection [41].

A limitation of the study is the approach used for the collection of data on risk factors used to determine how HPV was acquired and maintained, since the statistical associations were made solely based on the information reported by women. Therefore, another limitation is that the study lacks an adequate comparison group.

## Conclusions

This study provides a regional estimate on the prevalence of HPV infection among Quilombola women. It highlighted the higher prevalence of HPV type 68. This virus type can contribute to an increase in cervical carcinogenesis related to specific HPV genotypes.

It is believed that the findings of this study can foster the development of strategies aimed at enhancing the health of the Quilombola women towards the prevention and management of specific HPV infection in view of the particularities of this group. Greater knowledge, on the part of women, on the mode of HPV acquisition, risk factors and the frequency of HPV infection in different age and population groups are crucial. This is because, such knowledge would contribute to a greater perception of their risk of developing cervical preneoplastic lesions and therefore influence their continued adherence to screening strategies. It may also encourage changes in HPV risk behavior and lifestyle.

## Abbreviations

ASC-H: atypical squamous cells cannot exclude high-grade squamous intraepithelial lesions
ASC-US: atypical squamous cells of undetermined significance
CC: Cervical cancer
CIN 1: Cervical Intraepithelial Neoplasm grade 1
DEPAT: Department of Pathology
DNA: deoxyribonucleic acid
HIV: Human Immunodeficiency Virus
HPV: Human Papillomavirus
HSIL: high-grade intaepithelial lesion
INCA: Brazilian National Cancer Institute
LSIL: low-grade squamous intraepithelial lesion
NIBA: Nucleum of Basic and Applied Immunology
PCCU: Cervical Cancer Control Program
PCR: polymerase chain reaction
PGMY 09/11: primers
STD: Sexually transmitted diseases
UCM: universal collection medium
UFMA: Federal University of Maranhão
VLP: virus-like particles
